# Tau protein as a diagnostic marker for diffuse axonal injury

**DOI:** 10.1371/journal.pone.0214381

**Published:** 2019-03-22

**Authors:** Keisuke Tomita, Taka-aki Nakada, Taku Oshima, Takayuki Motoshima, Rui Kawaguchi, Shigeto Oda

**Affiliations:** Department of Emergency and Critical Care Medicine, Chiba University Graduate School of Medicine, Chiba, Japan; University of Florida, UNITED STATES

## Abstract

**Background:**

Diffuse axonal injury (DAI) is difficult to identify in the early phase of traumatic brain injury (TBI) using common diagnostic methods. Tau protein is localized specifically in nerve axons. We hypothesized that serum level of tau can be a useful biomarker to diagnose DAI in the early phase of TBI.

**Methods & results:**

We measured serum tau levels in 40 TBI patients who were suspected of DAI within 6 hours after TBI to evaluate the accuracy of the tau level as a diagnostic marker for DAI. Diagnosis of DAI was confirmed according to magnetic resonance imaging (MRI) findings. The serum tau level in the DAI group (n = 13) was significantly higher than that in the non-DAI group (n = 27) (DAI vs. non-DAI, 25.3 [0 to 99.1] pg/mL vs. 0 [0 to 44.4] pg/mL, *P* = 0.03)). A receiver-operating characteristic curve to evaluate the diagnostic ability of serum tau level within 6 hours for DAI showed an area under the curve of 0.690 with 74.1% for sensitivity and 69.2% for specificity. Serum tau level was not significantly higher in unfavorable outcome group (Glasgow Outcome scale [GOS] score = 1–3 at hospital discharge) compared with favorable outcome group (GOS score = 4–5) (*P* = 0.19).

**Conclusions:**

Tau protein may be a useful biomarker for diagnosis of DAI in the early phase of TBI.

## Introduction

Diffuse axonal injury (DAI) is a severe form of traumatic brain injury (TBI) caused by rotational or acceleration forces of the head, resulting in a shear disruption of the axons [1]. DAI is associated with poor prognosis due to the lack of specific treatment [2]: 15% resulting in vegetative state or significantly impaired cognitive function, with mortality over 40%. Yet, early diagnosis may provide chances for developing early treatment strategies, proven to be effective in stroke and other neurologic injuries [3]. DAI could be verified pathologically [4, 5]; however, it is difficult to be identified by the most commonly conducted examination for the initial diagnosis of TBI, namely computed tomography (CT) [6]. Magnetic resonance imaging (MRI) is recommended for diagnosing DAI [7, 8]; however it is often not feasible in the early phase of trauma care, requiring a long examination time in an isolated chamber with limited patient access and monitoring [9, 10]. Therefore, it is difficult to identify DAI using imaging diagnostic methods in the early phase of TBI.

Biomarkers can provide an option for early diagnosis of DAI and predict its prognosis [3]. Tau protein have been known to increase in concussed ice hockey players as a useful biomarker to predict their neurological outcome [11]. Since tau protein is localized specifically in neuronal axons [12], it is a potential biomarker for diagnosis of DAI. However, the serum tau level of patients who are suspected of DAI has not been studied to date.

We conducted the current study to test the hypothesis that serum level of tau protein is a useful biomarker to diagnose DAI in the early phase of TBI. We measured serum level of tau protein in patients who had TBI with disturbance of consciousness within 6 hours after injury and compared the results with the subsequent neurologic outcome.

## Materials and methods

### Patients

The prospective observational study was conducted in three tertiary critical care centers in Japan (Chiba University Hospital, Kimitsu Chuo Hospital and Senshu Trauma and Critical Care Center). The central institution of this study was Chiba University Hospital. The institutional review board of all institutions approved the study protocol (the institutional review board at Chiba University Hospital [approval number 1859], the institutional review board at Kimitsu Chuo Hospital [approval number 225] and the institutional review board at Senshu Trauma and Critical Care Center [approval number 592], respectively). All patients aged 20 years and older who admitted to the three centers for TBI with disturbance of consciousness between August 2014 and August 2016 were screened for eligibility. Exclusion criteria were as follows: (1) unavailability of blood sample, (2) lack of MRI, (3) absence of informed consent, (4) patients with neurologic disabilities (head injury, cerebral infarction, and hemorrhage) prior to the injury, (5) patients who arrived the hospital more than 6 hours after injury, (6) patients with cardiac arrest, or spinal cord injury. Written informed consent was obtained from all patients or their authorized representatives. Blood samples were taken promptly within 6 hours from injury. Samples were centrifuged for 30 minutes at 3000g and stored at -80°C until assayed according to the instructions of ELISA kit.

### Definitions

We defined DAI as TBI patients with high intensity area in corpus callosum, brain stem and gray-white matter junction of the cerebrum with T2-weighted imaging (T2WI), T2-weighted fluid attenuated inversion recovery (T2 FLAIR) and diffusion-weighted imaging (DWI) in magnetic resonance imaging (MRI) regardless of the level of consciousness [13–16]. The mass lesion was defined as high- or mixed- density lesion of more than 25cm^3^ [13]. The CT and MRI findings were independently evaluated by two physicians, a neurosurgeon and a radiologist, who had access to clinical information but were blinded to the serum tau protein level of the patients. In addition, head CT images were classified by the Helsinki CT score [17].

Clinical outcome was evaluated according to the Glasgow Outcome Scale (GOS) at the time of hospital discharge. The patient’s physical condition and willingness to resume normal occupational and social activities were taken into consideration. GOS is categorized as good recovery (GOS: 5), moderate disability (GOS: 4), severe disability (GOS: 3), vegetative status (GOS: 2), and death (GOS: 1). GOS 1–3 was defined as favorable neurological outcome and GOS 4–5 as unfavorable neurological outcome.

### Statistical analysis

We tested for differences in baseline characteristics using a Fisher`s exact test for categorical data and a Mann-Whitney’s U-test for continuous data. The primary outcome variable was presence of DAI. The primary analysis was the receiver-operating characteristic (ROC) curve analysis for the diagnostic accuracy of serum tau protein for identifying DAI within 6 hours from injury and the area under the curve (AUC) was calculated. Secondary outcome variable was neurological outcome at hospital discharge. Patients with favorable neurological outcome were compared with patients with unfavorable neurological outcome. The level of significance was set at α = 0.05 with a two-tailed test. All statistical analysis was performed with the GraphPad Prism 7 (GraphPad Software, San Diego, CA, USA).

## Results

**Fig 1:**
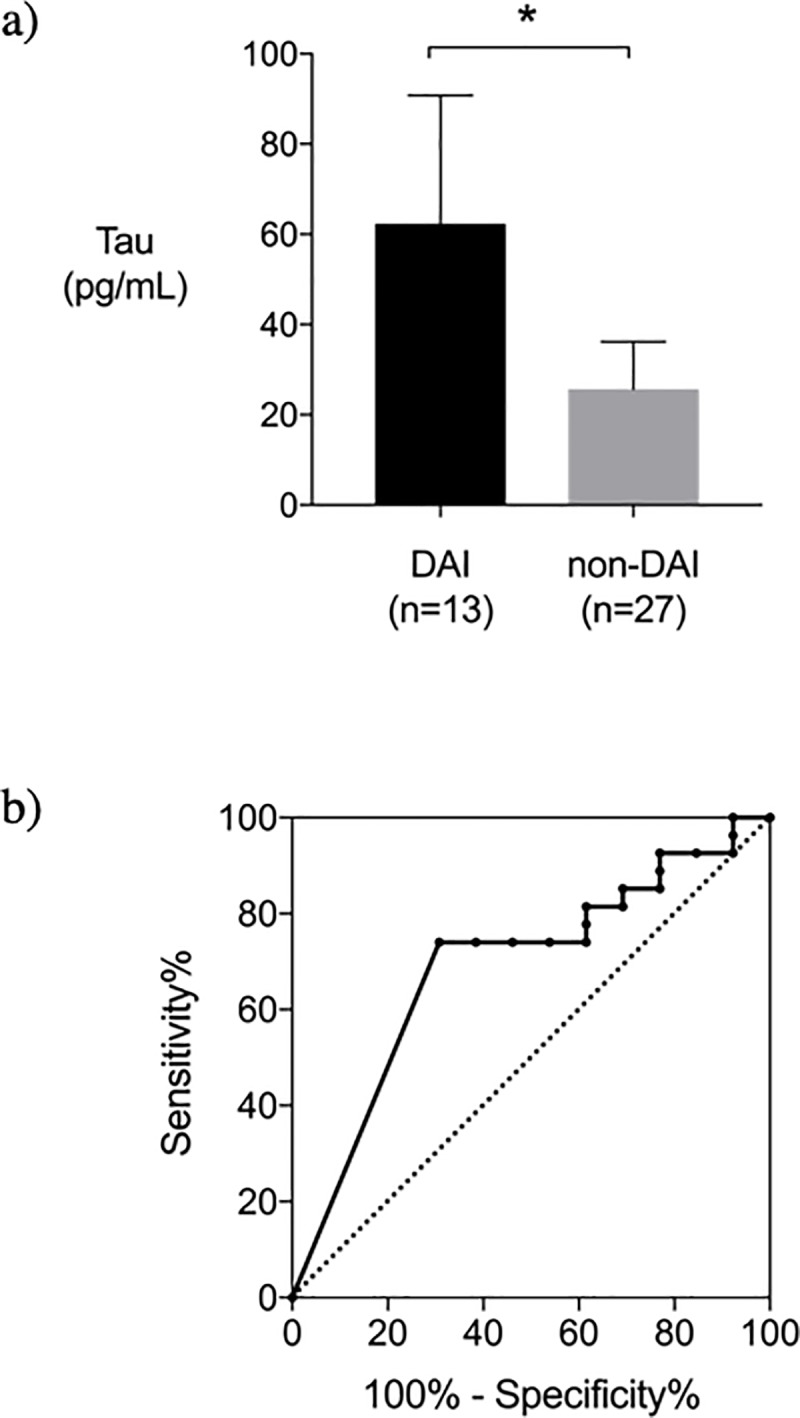
a) Serum tau protein levels within 6 hours from injury (diffuse axonal injury group vs. non-diffuse axonal injury group). Serum tau protein levels of diffuse axonal injury group were significantly higher than non-diffuse axonal injury group (*P* = 0.02). *P* values were calculated using Mann-Whitney’s U-test. Error bars indicate inter-quartile range. *P<0.05. b) Receiver Operating Characteristic Curve for serum tau protein within 6 hours from injury for diffuse axonal injury diagnosis. The area under the curve (AUC) was 0.690 (95% CI 0.512–0.867) with 74.1% sensitivity and 69.2% specificity at a serum tau protein level of 1.5 pg/mL.

Fifty-four TBI patients were enrolled in study period. Fourteen patients were excluded due to lack of MRI, and a total of 40 patients were analyzed. Thirteen patients were classified as DAI group and 27 patients as non-DAI group. Patients in the DAI group had significantly lower Glasgow Coma Scale on admission and higher Helsinki CT score (Table 1). Although the 28-day mortality was not different between DAI group and non-DAI group, GOS at hospital discharge of DAI group was lower than that of non-DAI group (Table 1). DAI group had significantly higher serum levels of tau protein compared to non-DAI group within 6 hours after injury (*P* = 0.02) (Fig 1A). The ROC curve analysis revealed AUC of 0.690 (95% confidence interval [CI], 0.512–0.867). The sensitivity and specificity of serum tau protein for diagnosing DAI were 74.1% and 69.2%, respectively, with a cut-off value of 1.5 pg/mL (Fig 1B**).** There was no significant difference in serum levels of tau protein between patients with and without subarachnoid hemorrhage (*P* = 0.45).

**Table 1:** Baseline characteristics of study patients with DAI and non- DAI.

Baseline characteristics and clinical outcome	DAI (n = 13)	Non-DAI (n = 27)	*P* value
Baseline characteristics			
Age -yr	65 (46–77)	46 (30–70)	0.14
Gender -% male	69.2	77.8	0.70
Initial Glasgow Coma Scale	8 (5.5–12.5)	14 (10–14)	0.003
Cause of injury -n (%)			
Traffic accident	12 (92.3)	14 (51.9)	0.01
Fall	1 (7.7)	7 (25.9)	0.24
Others	0 (0)	6 (22.2)	0.15
Anisocoria -n (%)	7 (53.9)	5 (18.5)	0.03
Abnormal pupil light reflex -n (%)	4 (33.3)	3 (12.0)	0.18
ISS except the head	4 (0.5–14.5)	4 (0–9)	0.59
Body temperature -°C	36.2 (36.0–36.4)	36.3 (35.8–36.7)	0.77
Mean blood pressure -mmHg	97.5 (78.3–100.0)	105.3 (97.8–115.3)	0.02
D-dimer -μg/mL	34.8 (9.1–79.6)	16.2 (4.2–27.3)	0.07
CT finding -n (%)			
Mass lesion	0 (0)	1 (3.7)	>0.99
Subarachnoid hemorrhage	11 (84.6)	13 (48.2)	0.04
Subdural hematoma	7 (53.9)	9 (33.3)	0.30
Epidural hematoma	2 (15.4)	3 (11.1)	>0.99
Intracerebral hematoma	10 (76.9)	9 (33.3)	0.02
Helsinki CT score	4 (1–4.5)	0 (0–2)	0.03
Clinical outcome			
28-day mortality -n (%)	1 (7.7)	0 (0)	0.33
GOS at hospital discharge	3 (3–3)	5 (3–5)	0.0005
Favorable outcome -n (%)	2 (15.4)	20 (74.1)	0.0007
Unfavorable outcome -n (%)	11 (84.6)	7 (25.9)	0.0007

ISS, Injury severity score

GOS, Glasgow Outcome Scale; Favorable outcome = GOS 4–5; Unfavorable outcome = GOS 1–3

Data are median and inter-quartile range for continuous variables. *P* values were calculated using a Fisher`s exact test for categorical data and a Mann-Whitney’s U-test for continuous data.

**Fig 2:**
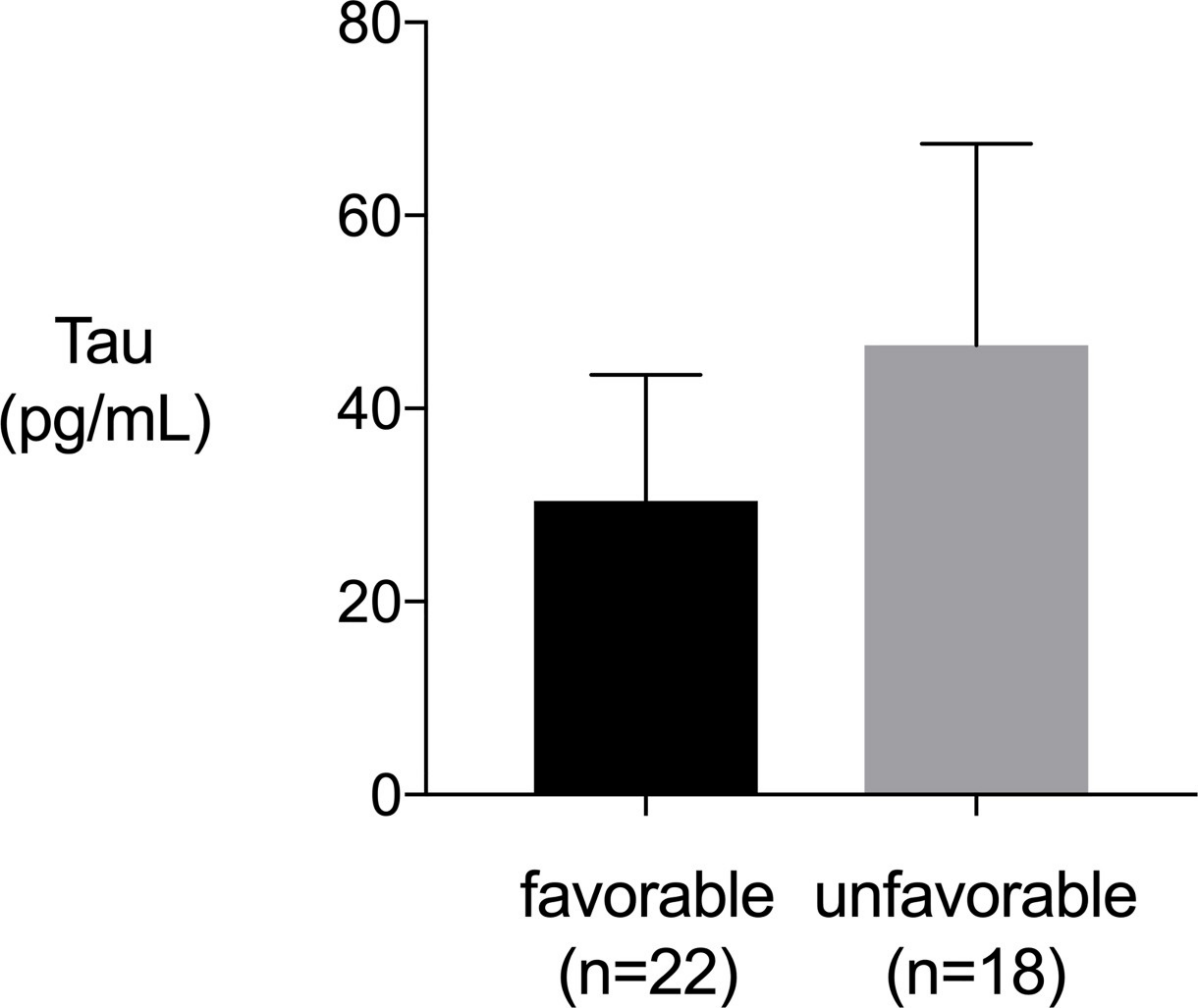
Serum tau protein levels within 6 hours from injury (favorable outcome group vs. unfavorable outcome group). Serum tau protein levels of unfavorable outcome group were not significantly higher than favorable outcome group (*P* = 0.19). *P* values were calculated using Mann-Whitney’s U-test. Error bars indicate inter-quartile range.

Patients who had unfavorable neurological outcome (GOS 1–3) were older age, lower initial GCS score and higher Helsinki CT score and DAI compared to the patients who had favorable outcome (GOS 4–5) (Table 2). The serum tau protein level was not different significantly between the unfavorable neurological outcome group and the favorable outcome group. (*P* = 0.19) (Fig 2)

**Table 2:** Baseline characteristics of study patients by the neurological outcome at hospital discharge.

	Favorable outcome (n = 22)	Unfavorable outcome (n = 18)	*P* value
Age -yr	42.5 (28.5–64.3)	68.5 (55.3–79)	0.006
Gender -% male	68.2	83.3	0.46
Initial Glasgow Coma Scale	14 (13–15)	9 (6–13)	<0.0001
Cause of injury -n (%)			
Traffic accident	12 (54.6)	14 (77.8)	0.19
Fall	5 (22.7)	3 (16.7)	0.71
Others	5 (22.7)	1 (5.6)	0.20
ISS except the head	4 (0–9)	6.5 (0.8–13.8)	0.33
Body temperature -°C	36.2 (35.8–36.9)	36.3 (36.0–36.4)	0.77
Mean blood pressure -mmHg	105.3 (96.8–110.6)	100 (89.7–117.2)	0.42
D-dimer -μg/mL	15.5 (3.4–21.9)	29.6 (10.0–49.4)	0.05
CT finding -n (%)			
Mass lesion	1 (4.5)	0 (0)	>0.99
Subarachnoid hemorrhage	8 (36.4)	16 (88.9)	0.001
Subdural hematoma	6 (27.3)	10 (55.6)	0.11
Epidural hematoma	3 (13.6)	2 (11.1)	>0.99
Intracerebral hematoma	7 (31.8)	12 (66.7)	0.05
Helsinki CT score	0 (0–2)	4 (0–4.3)	0.02
Diagnosis of DAI -n (%)	2 (9.1)	11 (61.1)	0.0007

DAI, diffuse axonal injury

Data are median and inter-quartile range for continuous variables. *P* values were calculated using a Fisher`s exact test for categorical data and a Mann-Whitney’s U-test for continuous data.

## Discussion

In the present study of serum levels of tau protein in the early phase of TBI patients with suspected DAI, serum levels of tau presented high diagnostic accuracy for DAI (AUC = 0.690; sensitivity, 74.1%; specificity, 69.2%).

Neurofilament (NF), an axon-specific biomarker, is also considered as the one of the useful diagnostic biomarkers of DAI [18]. In a study measuring serum level of NF in 9 patients suspected of DAI because of affected consciousness and/or focal neurological symptoms without an obvious explanation seen on the CT scan of the brain, serum NF concentration of the patients with DAI were significantly higher than serum levels in healthy controls. However, since the time point of blood sampling was not specified, the usefulness of NF for the early diagnosis of DAI could not be evaluated [19]. S-100 calcium-binding protein B (S-100B) and neuron specific enolase (NSE) have been studied as a potential biomarker to predict neurologic outcome or mortality in the initial phase of trauma care, but with unsatisfactory results [20, 21]. The fact that these molecules exist in tissues outside of the brain poses a serious limitation that blood levels of these biomarkers could be elevated in trauma patients without TBI [22, 23].

DAI is the widespread damage of axons in white matter of the brain, resulting from shear strain forces caused by marked rotational acceleration and deceleration of the head during an impact [5]. Tau protein is a microtubule associated protein with a molecular weight of 48 kDa to 67 kDa and has highly specific expression in neuronal axons. Tau protein assembles axonal microtubule bundles and these bundles are important structural elements in the axonal cytoskeleton [12]. Animal study using rat focal brain injury model revealed higher serum tau levels compared to sham operated controls, recording the highest level at 1 hour after injury compared to values at 6h, 24h, 48h, and 168h. Serum level of tau increased according to the severity of the injury (sham vs. mild, *P*<0.001; mild vs. severe, *P*<0.001) [24]. In a study of 15 head trauma patients, cleaved tau protein levels in cerebrospinal fluid (CFS) were more than 1000 times higher than neurologic or non-neurologic (psychiatric) controls (1519 ± 3019 vs. 0.03 ± 0.1 or 0 ± 0 ng/mL, respectively) [25]. The significance of tau is fairly well studied in sports related head trauma. An investigation of CFS samples from 30 Olympic boxers 1–6 days after bouts revealed significant elevation of tau protein compared to CSF samples of their healthy peer controls (58 ng/L vs. 45 ng/L, P = 0.025) [26]. In a study enrolling 288 professional hockey players, tau levels increased significantly after brain concussion (4.5 [0.06 to 22.7] pg/mL vs. 10.0 [2.0 to 171) pg/mL, P<0.001), and presented higher diagnostic accuracy compared with other known biomarkers of brain injury, namely S-100B and NSE (AUC: tau = 0.80, S100 = 0.67, NSE = 0.55) [11]. The higher tau levels in the TBI patients of our cohort compared with the hockey players is likely to be attributed to the severity of the injury; tau levels were significantly elevated in DAI patients within 6 hours of injury compared with non-DAI controls (25.3 [0 to 99.1] pg/mL vs. 0 [0 to 44.4] pg/mL, *P* = 0.03). The serum levels of tau also presented relatively high diagnostic accuracy (AUC = 0.690; sensitivity, 74.1%; specificity, 69.2%).

The usefulness of tau protein for predicting neurologic outcome of traumatic brain injury has been evaluated in a study enrolling 56 patients by measuring the serum level of tau serially (12h, 1d, 2d, 3d, 4d, 7d, 14d) after injury and analyzing the association with extended Glasgow outcome scale (GOSE). Serum level of tau on the 2^nd^ day after injury presented the strongest associated with the neurologic outcome; mean tau values for favorable outcome group (GOSE 1–4) was significantly higher than the unfavorable outcome group (GOSE 5–8) (341.4±29.3 pg/ml vs. 72.9±8.9, p<0.0001) [27]. Also in a study measuring serum tau protein levels in 34 severe TBI patients, patients who had poor neurological outcome (GOS 1–3 at 6 month post- injury) had significantly increased tau levels compared to those with good neurological outcome (GOS 4–5) (serum tau levels; poor outcome [436.2 ± 473.6 pg/mL] vs. good outcome [51.6 ± 81.5 pg/mL]). In accord with this, we found that patients with poor neurological outcome had increased tau levels compared to those with good neurological outcome (serum tau levels; poor outcome [104.7 ± 215.5 pg/mL] vs. good outcome [29.7 ± 59.0 pg/mL]) [28].

There are several limitations in the present study. First, the sample size was relatively small to evaluate the diagnostic value of tau protein for the severity of DAI. Second, although we evaluated the neurological outcome with GOS at hospital discharge as previously described, it might not have been sufficient for the evaluation of their long-term outcomes [29, 30]. However, this is the first multicenter, prospective study to evaluate the diagnostic accuracy of serum tau protein levels for identifying DAI in early phase of TBI. Further large-scale study is warranted to evaluate the clinical usefulness of tau protein for diagnosis of DAI.

In conclusion, serum tau protein levels may be useful as a diagnostic biomarker for DAI within 6 hours after injury.
